# Localisation of Human Papillomavirus 16 E7 Oncoprotein Changes with Cell Confluence

**DOI:** 10.1371/journal.pone.0021501

**Published:** 2011-06-29

**Authors:** Joanna Laurson, Kenneth Raj

**Affiliations:** Division of Virology, National Institute for Medical Research, Medical Research Council, London, United Kingdom; Karolinska Institutet, Sweden

## Abstract

E7 is one of the best studied proteins of human papillomavirus type 16, largely because of its oncogenic potential linked to cervical cancer. Yet the sub-cellular location of E7 remains confounding, even though it has been shown to be able to shuttle between the nucleus and the cytoplasm. Here we show with immunocytochemistry that E7 proteins are located in the nucleus and cytoplasm in sub-confluent cells, but becomes cytoplasmic in confluent cells. The change in E7's location is independent of time in culture, cell division, cell cycle phase or cellular differentiation. Levels of E7 are also increased in confluent cells as determined by Western blotting. Our investigations have also uncovered how different analytical techniques influence the observation of where E7 is localised, highlighting the importance of technical choice in such analysis. Understanding the localisation of E7 will help us to better comprehend the function of E7 on its target proteins.

## Introduction

Human papillomavirus 16 (HPV16) is one of the most prevalent high-risk HPV types associated with cervical cancer [1], [2]. The two HPV16 oncoproteins E6 and E7 are able to immortalise keratinocytes [3] and other cell types due to their ability to alter the levels of various cellular proteins that control cellular proliferation. The HPV16E6 protein is able to direct p53 protein for degradation [4], [5] and stimulate expression of the catalytic subunit of telomerase, hTERT [6], [7]. The HPV16E7 protein's most prominent activity is binding to the tumour suppressor protein pRb [8]. Hence it is not surprising that the E7 protein of HPV16 is one of the best studied proteins of the virus. In spite of this, the location of this protein within the cell is still unclear.

Previous studies investigating the intracellular localisation of HPV16E7 protein have been equivocal. E7 has been reported by different groups to be found predominantly in the cytoplasm [9], [10], nucleus [11], [12] or both in the nucleus and cytoplasm [13]–[15]. A recent study also reported a combination of different localisation of E7 in the same population of cells [16]. The ability of E7 to be in the nucleus and/or cytoplasm is by itself not surprising as it possesses both nuclear import and export signals, thus allowing it to shuttle between the two compartments [17], [18]. However, we are still uninformed in regards to the cellular circumstances that influence the location of E7 protein in the cell. To address this question, we used four cell lines, two of which were derived from naturally occurring cancers that contained integrated copies of HPV16 DNA (SiHa [19] and CaSki [20]), one derived from a pre-cancerous lesion containing episomal HPV16 DNA (W12) [21] and a non-tumorigenic foreskin keratinocyte cell line NIKS [22], into which episomal HPV16 DNAs were introduced [23]. Our analyses revealed that the localisation of the E7 protein is profoundly influenced by cell confluence.

## Results

### E7 localises into the cytoplasm in confluent cells

**Figure 1:**
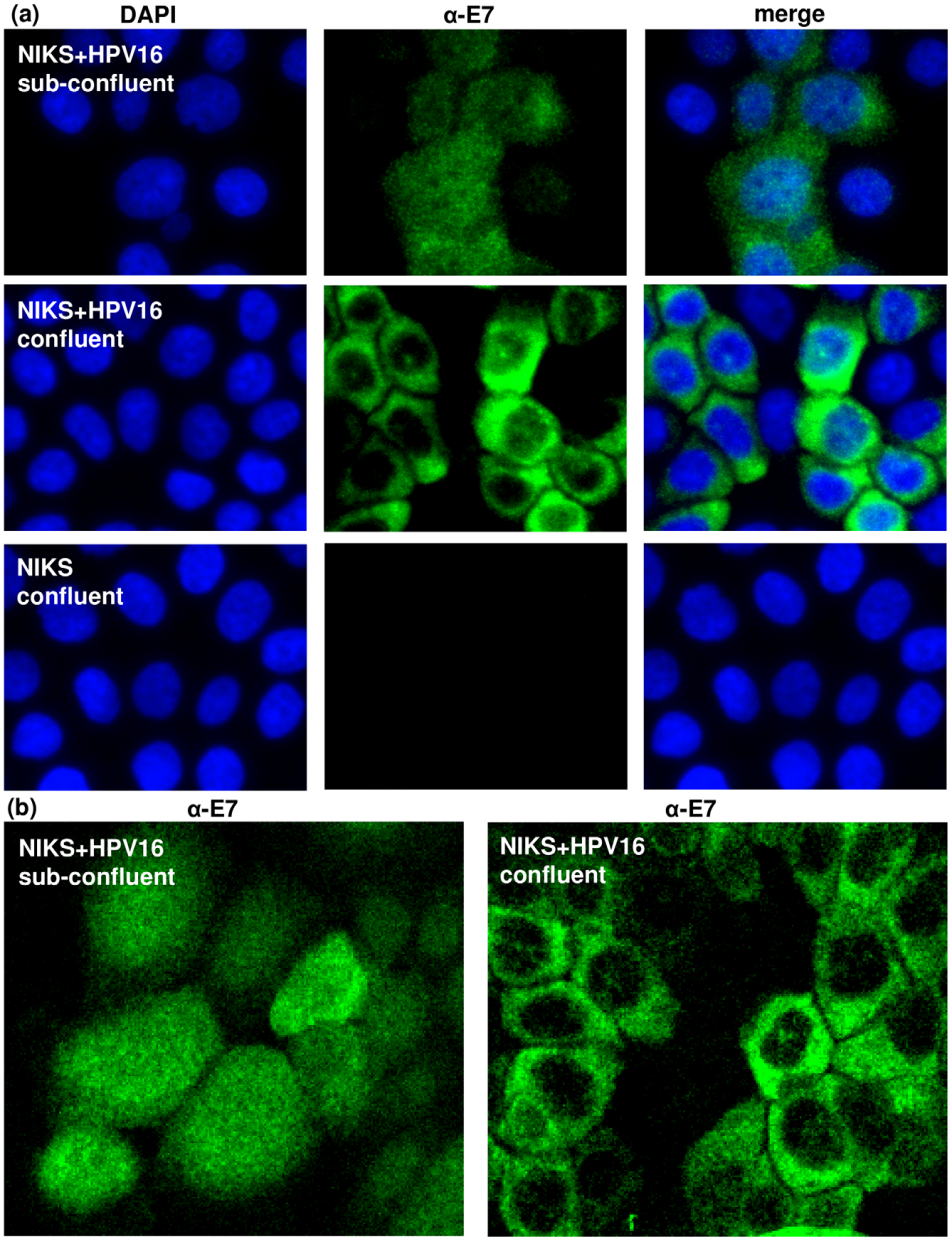
E7 expressed from episomes localises in the cytoplasm in confluent cells. (a) Immunofluorescence of HPV16E7 protein in sub-confluent and confluent NIKS+HPV16 cells. NIKS cells (with no HPV16) were analysed in parallel as a negative control. (b) Confocal microscopy of HPV16E7 protein in sub-confluent and confluent NIKS+HPV16 cells.

We have previously reported the generation of cell lines from NIKS cells that stably harbour episomal HPV16 DNA [23]. The viral DNA in these cells is active and they express numerous viral proteins including the E7 protein. Immunocytochemical staining of these cells (NIKS+HPV16) revealed that while E7 was present in the nucleus and the cytoplasm when the cells were sub-confluent, its location became predominantly cytoplasmic when the cells were confluent (Figure 1a–b). Confluence was defined by cell number; sub-confluent cultures <9×10^4^ cells/cm^2^ and confluent cultures >3.1×10^5^ cells/cm^2^. This definition is strictly adhered to at all times and is implicit in all description in this report.

**Figure 2:**
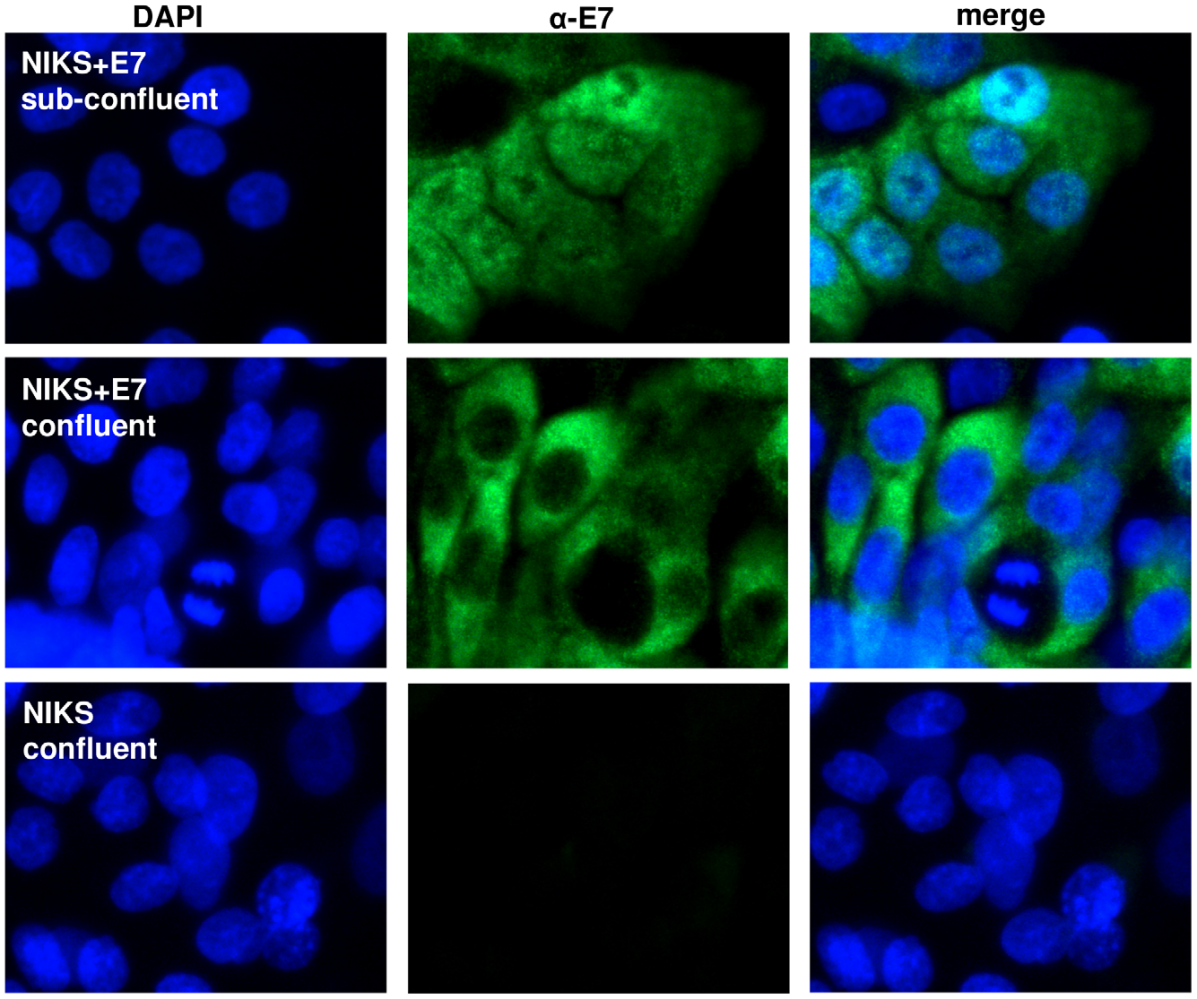
E7 expressed alone localises in the cytoplasm in confluent cells. Immunofluorescence of HPV16E7 protein in sub-confluent and confluent NIKS+E7 cells (only expressing E7). NIKS cells (vector control) were analysed in parallel as a negative control.

To address the possibility that the switch of localisation may be mediated by interaction between E7 and other proteins that are expressed from the viral episomes, we generated NIKS cell lines that only expressed the HPV16E7 protein. We infected NIKS cells with retroviruses bearing the HPV16E7 gene (LXSN16E7) and staining of these cells (NIKS+E7) revealed that the E7 protein was present in the nucleus and cytoplasm when the cells were not confluent but it became strongly cytoplasmic upon cell confluence, demonstrating that this phenomenon is independent of other HPV proteins (Figure 2).

**Figure 3:**
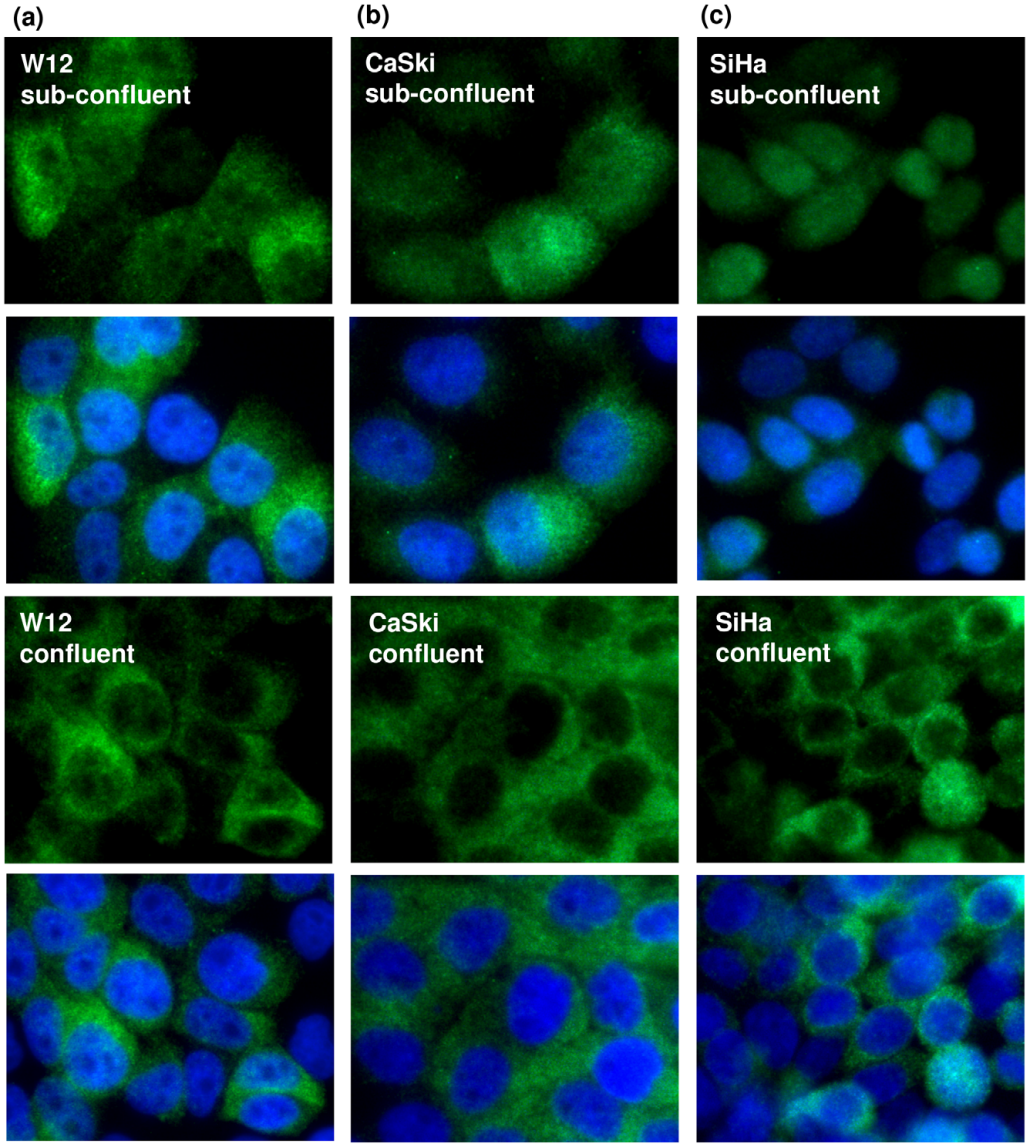
E7 in cell lines derived from a pre-cancerous lesion and naturally occurring cancers localises in the cytoplasm in confluent cells. Immunofluorescene of HPV16E7 protein in sub-confluent and confluent (a) W12, (b) CaSki and (c) SiHa cells.

To determine if this phenomenon was specific to NIKS cells, which were derived from foreskin, we stained for the E7 protein in W12 cells. W12 cells were originally derived from a low-grade cervical lesion and harbour episomal HPV16 DNA [21]. Again, we observed the confluence-dependent localisation of E7 proteins (Figure 3a). This results demonstrate that the switch in E7 localisation is independent of the gender of the cells and the tissue from which these cells were derived (foreskin and cervix). However, a common feature between NIKS and W12 cells is that they were not derived from cancerous tissues. To test if this oncoprotein's confluence-dependent relocalisation also occurs in cancer cells, tumour cell lines CaSki [20] and SiHa [19], which contain integrated copies of HPV16 DNA, were subjected to similar (confluent and non-confluent) growth conditions and stained for E7 proteins. We found that the E7 protein in these cells also behaved in the same way as those in NIKS and W12 cells (Figure 3b–c). These results confirmed that this phenomenon is neither cell-type nor cell state (transformed or not) specific. It is interesting to note that E7 levels were heterogeneous between cells in a population. While this could be due to cells possessing varied copy numbers of episomal HPV16 DNA (as in the case of W12), it is clear that other unknown factors must also be involved, as SiHa and CaSki cells (with integrated HPV16 DNA) also exhibited heterogenous E7 protein levels between cells of the same population.

The relocation of E7 to the cytoplasm was observed irrespective of whether cell confluence was attained by culturing the cell for several days longer after non-confluent cells were collected for analyses, or seeding different amounts of cells in separate dishes in order to attain either non-confluent or confluent (as numerically defined above) populations the same number of days after seeding. This demonstrates that the reduction of E7 in the nucleus of confluent cells is not influenced by trypsinisation, nutrient and growth factor availability during media changes or by time in culture.

**Figure 4:**
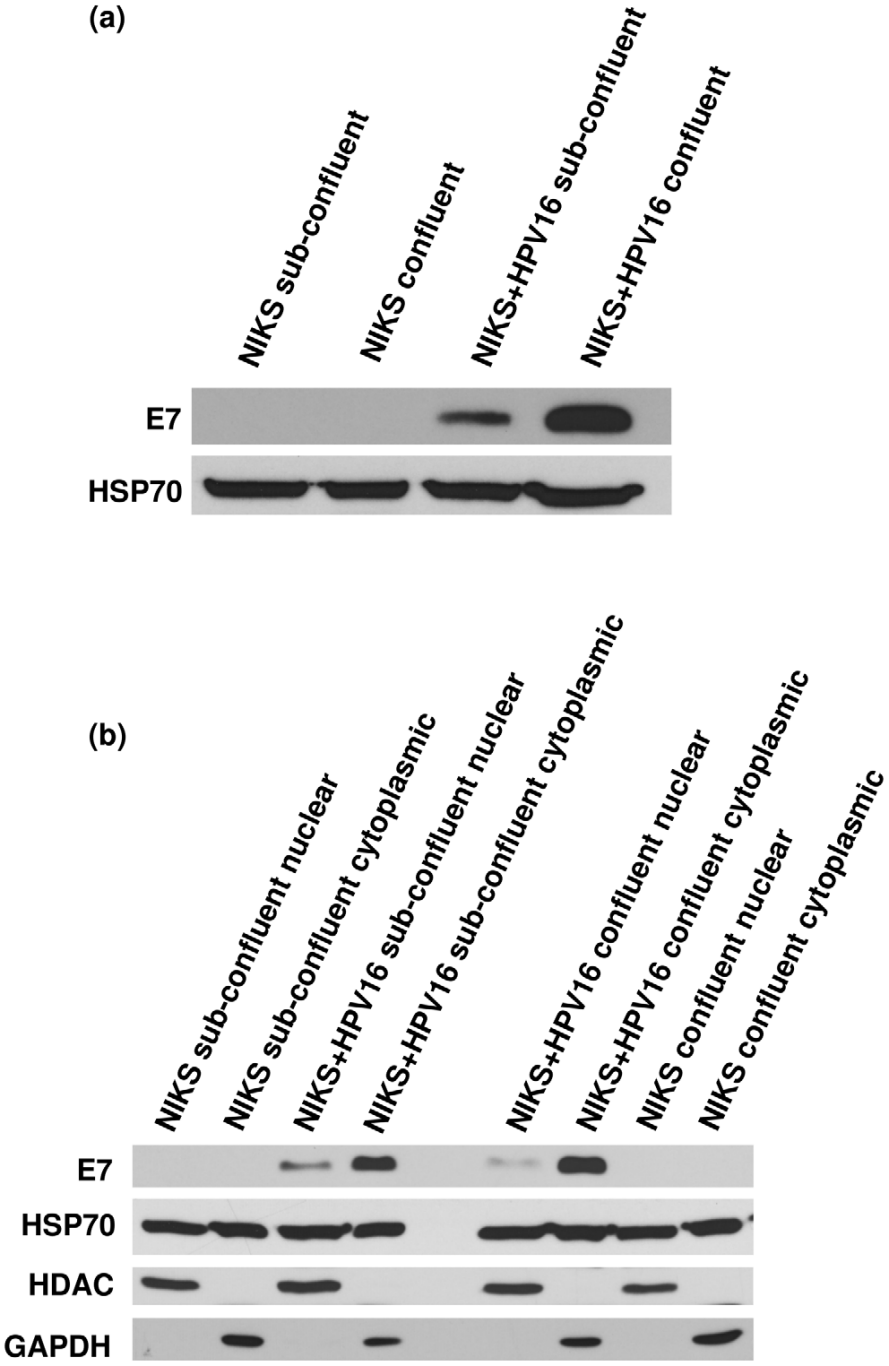
Levels of E7 increase in confluent cells. Western blotting of HPV16E7 protein in sub-confluent and confluent NIKS and NIKS+HPV16 using (a) whole cell lysates and (b) nuclear and cytoplasmic fractions. HSP70 used as a loading control and HDAC2 and GAPDH as fractionation controls.

The analyses carried out thus far have been immunocytochemical staining of the E7 protein. While this is undoubtedly the best way to ascertain the location of proteins *in situ*, it does not show us the relative quantities of the E7 proteins in the different populations of cells. Immunoblotting of whole cell extracts showed levels of E7 increase in confluent NIKS+HPV16 cells (Figure 4a). What is perplexing however, is that when the cells were fractionated to nuclear and cytoplasmic lysates, E7 protein appeared predominantly cytoplasmic in both cell populations (Figure 4b). This was repeatedly observed using different fractionation protocols. The balance of relative quantities of cytoplasmic and nuclear E7 did not reflect the *in situ* situation that was revealed through immunocytochemical staining. This curious feature has been noted by others, for example Smith-McCune *et al.* who commented that while Western blotting-fractionation analyses show E7 to be cytoplasmic, immunocytochemical analyses demonstrated E7 to also be nuclear, prompting the suggestion that E7 leaks out of the nucleus during fractionation [24], [25]. This suggestion is consistent with our observations and highlights another confounding factor that influences the results in this area of research.

### Blocking nuclear export inhibits cytoplasmic localisation of E7 in confluent cells

**Figure 5:**
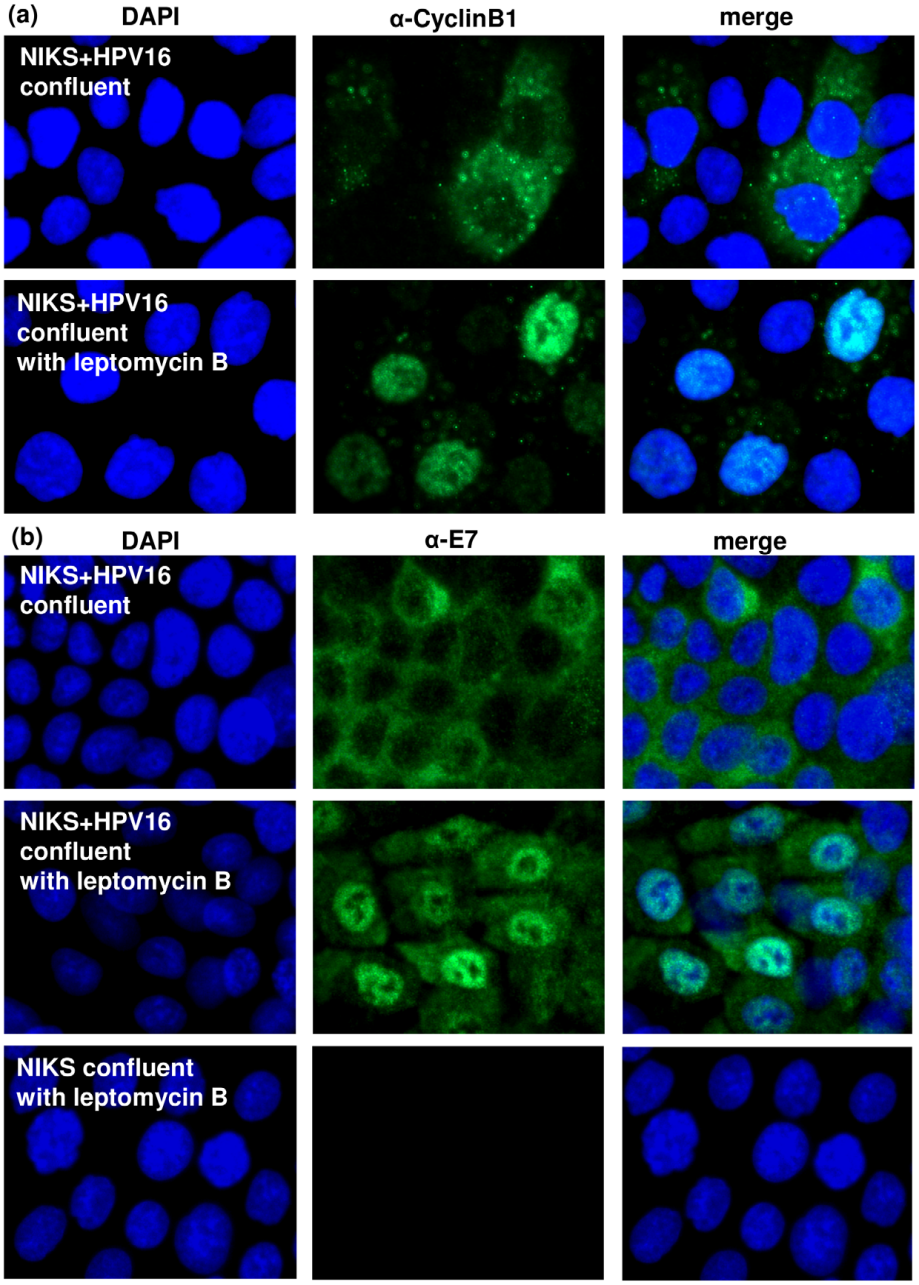
Blocking nuclear export inhibits cytoplasmic localisation of E7 in confluent cells. (a) Immunofluorescence of cyclin B1 used as a positive control for nuclear export inhibition in confluent NIKS+HPV16 cells treated with leptomycin B. (b) Immunofluorescence of HPV16E7 protein in confluent NIKS+HPV16 cells treated with leptomycin B to block nuclear export. NIKS control cells (with no HPV16) treated with leptomycin B were analysed in parallel.

It has been suggested that the E7 protein forms spherical oligomers in the cytoplasm [26]. It is possible that in confluent cells, the E7 proteins are held strongly in the cytoplasm in such oligomeric structures, which may be static. To determine whether E7 proteins form a static cytoplasmic presence in confluent cells, we treated NIKS+HPV16 cells with leptomycin B, which inhibits nuclear export of proteins by exportin-1 [27]. Leptomycin B effectively prevented cyclin B in these cells from exiting the nucleus and as a consequence cyclin B, which is normally a cytoplasmic protein, became almost exclusively nuclear (Figure 5a). Analyses of the E7 protein in these confluent cells revealed that inhibition of nuclear export by leptomycin B has also caused the E7 protein to become predominantly nuclear (Figure 5b); suggesting that the cytoplasmic presence of E7 is not static, and that an active and constant export of E7 into the cytoplasm is necessary to keep E7 predominantly cytoplasmic in confluent cells. It is perhaps of interest to note that while leptomycin B excluded cyclin B from the cytoplasm almost completely, a certain amount of E7 protein remained in the cytoplasm of these cells. It is possible that a small but detectable proportion of cytoplasmic E7 protein might actually be static. This however does not detract from the fact that the vast majority of E7 proteins in confluent cells require constant nuclear export to retain them in the cytoplasm.

### Cytoplasmic localisation of E7 is not linked to the cell cycle

**Figure 6:**
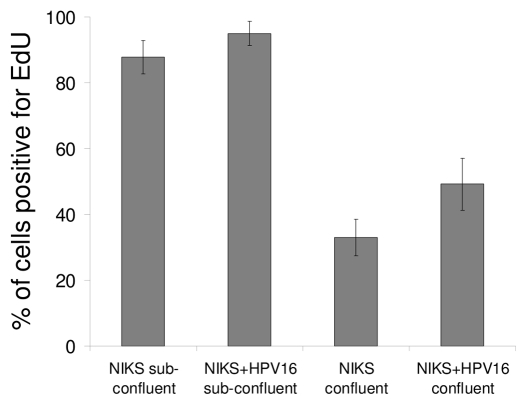
Localisation of E7 is not linked to cellular proliferation. Sub-confluent and confluent NIKS and NIKS+HPV16 cells were treated with EdU (BrdU alternative) to label cells undergoing DNA synthesis. The cells were stained for EdU and the percentage of labelled cells was calculated.

As confluence is often associated with cell cycle arrest, we tested whether the confluence-dependent cytoplasmic localisation of the E7 protein was coupled to the termination of cell proliferation. To determine the proliferative state of confluent cultures of NIKS+HPV16 cells, we labelled sub-confluent and confluent cells with EdU (alternative to BrdU), which is a nucleotide analogue and is incorporated into DNA-replicating cells. Even though there was a reduction in the number of replicating cells in confluent cultures compared to sub-confluent ones, virtually half (49%) of all the confluent cells were still proliferating (Figure 6). This figure contrasts with that of confluent cells that had predominantly cytoplasmic E7 protein in at least 80% of the cells (Figure 1); suggesting that the localisation of E7 to the cytoplasm is not coupled to cessation of cellular proliferation.

**Figure 7:**
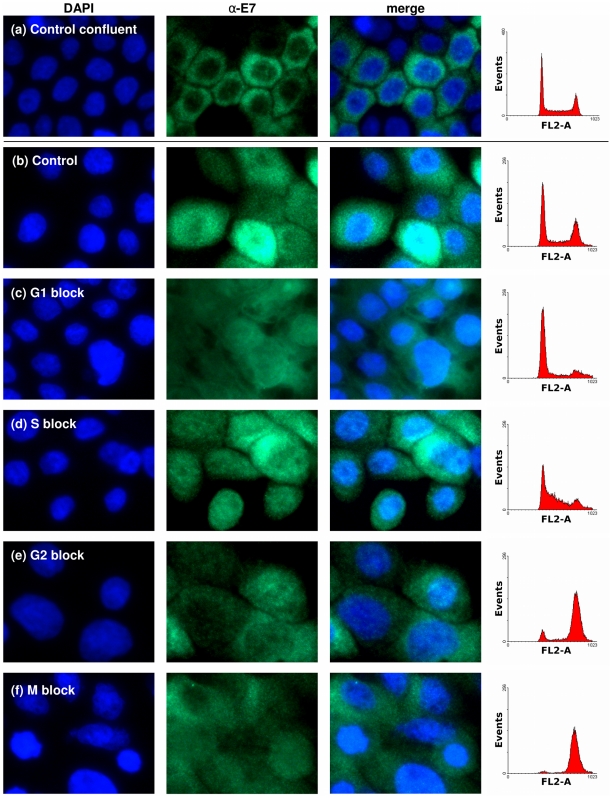
Cytoplasmic localisation of E7 is not linked to the cell cycle. Immunofluorescence of HPV16E7 protein in sub-confluent NIKS+E7 treated with different compounds to arrest the cell cycle at different stages. (a) Confluent control (b) untreated sub-confluent control and sub-confluent (c) mimosine (G1 block), (d) thymidine (S-phase block), (e) etoposide (G2 block) and (f) nocodazole (mitosis block). Cell cycle blocks were confirmed in parallel by propidium iodide staining analysed by flow cytometry.

Next we investigated if the cytoplasmic localisation of E7 was associated with a particular phase of the cell cycle. NIKS+HPV16 cells were arrested at G1, S, G2 or mitosis with mimosine, thymidine, etoposide and nocodazole respectively. The blocked states of these cells were confirmed by their DNA content through flow cytometry. Staining for the E7 proteins in these cells shows that none of the sub-confluent drug-arrested cells at any of the phases have E7 localised predominantly to the cytoplasm as observed in confluent cells (Figure 7).

Confluent keratinocytes are known to undergo some form of early stage differentiation. To test whether early-stage cellular differentiation was responsible for E7's confluence-induced cytoplasmic localisation, we induced differentiation of sub-confluent NIKS-HPV16 cells with high calcium together with growth factor withdrawal. While this did indeed stimulate early-stage differentiation of the sub-confluent cells, as evidenced by cytokeratin 10 expression, E7 did not re-localise to the cytoplasm as they do in confluent cells (data not shown).

Collectively, these results show that while the cytoplasmic localisation of E7 is linked to cell confluence, it is not due to the cessation of proliferation, the arrest at a particular cell cycle phase or differentiation of these confluent cells. Instead it appears that a yet-to-be elucidated mechanism triggered by greater cell-cell contact between confluent cells underlies the cause for the cytoplasmic localisation of E7.

### Targets of E7 at sub-confluence and confluence

**Figure 8:**
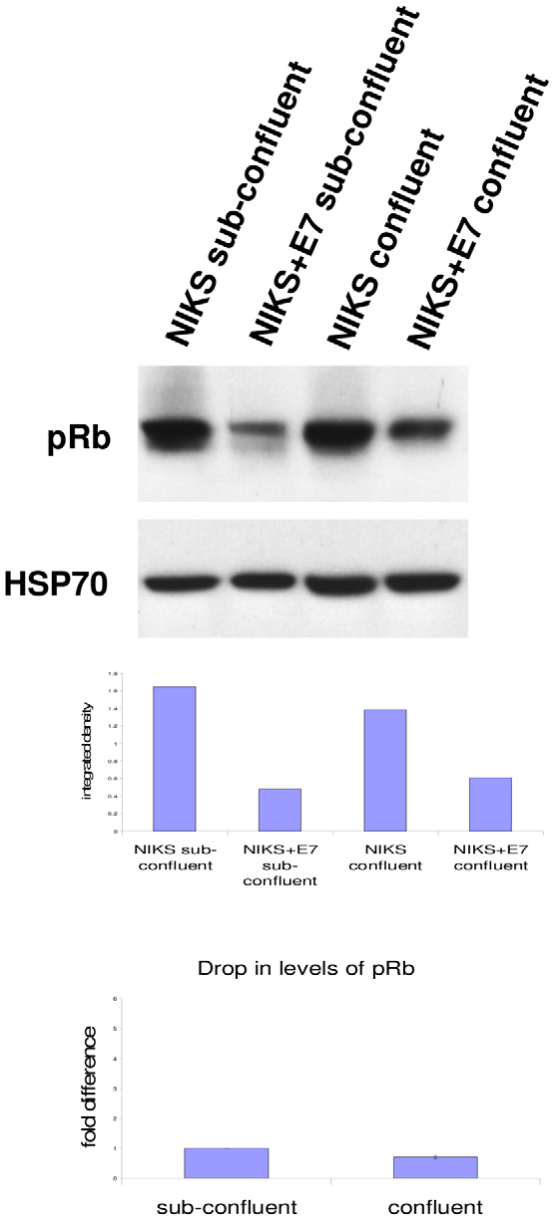
Effect of E7 on pRb at sub-confluence and confluence. Western blot analysis of pRb in sub-confluent and confluent NIKS and NIKS+E7 cells. HSP70 is shown as a loading control. Graphs show integrated density measured by ImageJ and normalised to HSP70 for the blots shown. Two replicates were analysed for the reduction of pRb and the error bars show the range of the data (i.e. the lowest and highest values).

**Figure 9:**
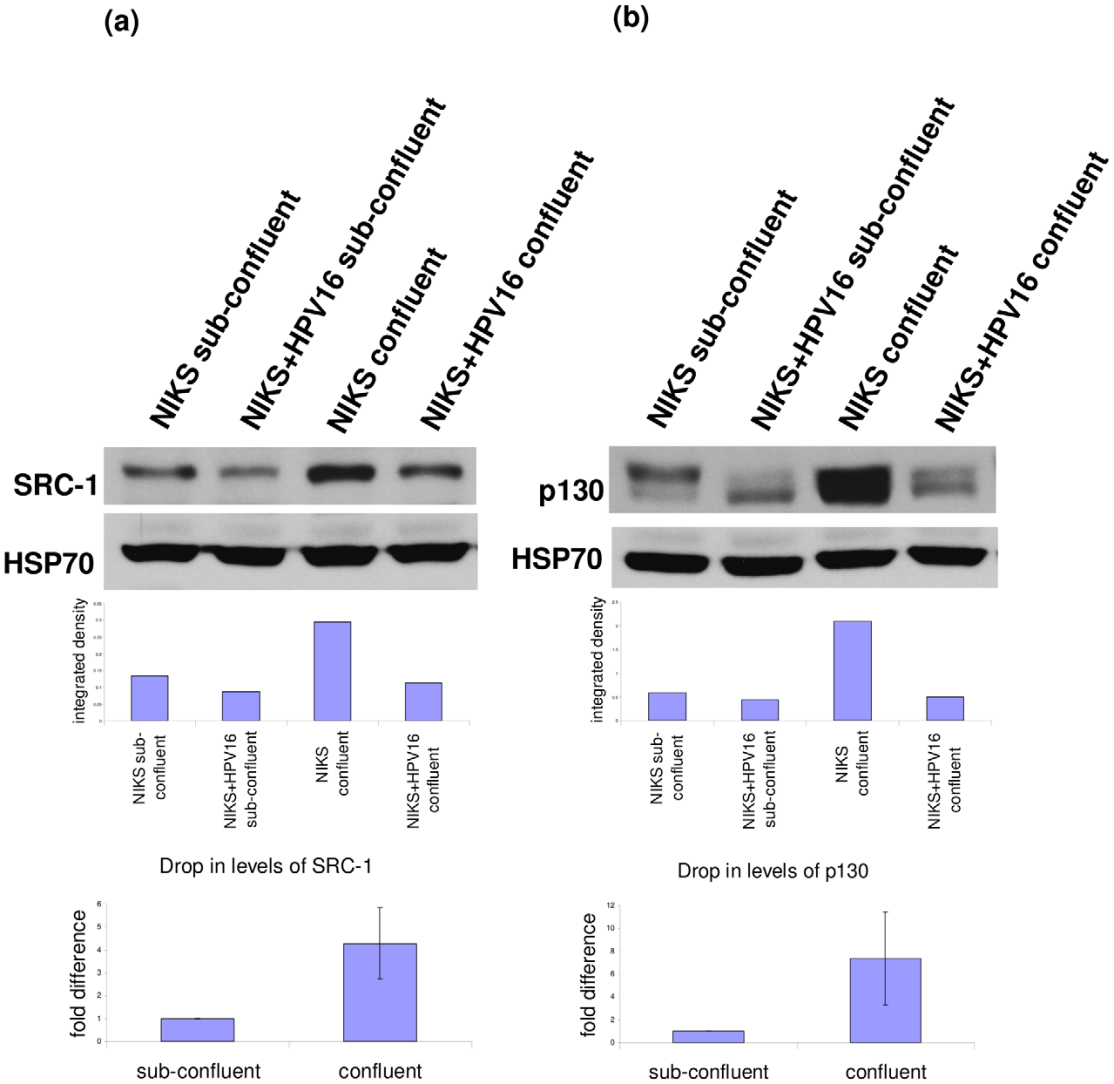
Effects of E7 on SRC-1 and p130 at sub-confluence and confluence. Western blot analysis of (a) SRC-1 and (b) p130 in sub-confluent and confluent NIKS and NIKS+HPV16 cells. HSP70 is shown as a loading control. Graphs show integrated density measured by ImageJ and normalised to HSP70 for the blots shown. Three replicates were analysed for the reduction of SRC-1 and p130 and the error bars show standard deviation. On average the drop in levels of SRC-1 was 4.37 fold more and p130 was 7.40 fold more in confluent cells compared to sub-confluent cells.

Next, we turned our attention to the effect of this phenomenon on E7's activity. We analysed the levels of three binding partners of E7; pRb [8], SRC-1 [28] and p130 [24], [29]. Regardless of E7's location, it was able to cause the reduction of these proteins (Figures 8 and 9). pRb levels were reduced to a similar extent in both confluent and sub-confluent cells (Figure 8). Interestingly, this was not the case for SRC-1 and p130 (Figure 9). In spite of the big increase in the levels of these proteins in confluent cells, E7 protein was still able to reduce their levels. This may be due to higher E7 levels, but it is also possible that the localisation of E7 to the cytoplasm is a contributing factor. Whatever the case may be, it is important to note that cell confluence has a large impact on the magnitude of reduction of some E7 targets such as SRC-1. SRC-1 was previously reported to be a binding partner of E7 but was not observed to be reduced by E7. However, when confluent cells were analysed, the reduction of SRC-1 was much more obvious and significant. Hence the effect of E7 on the protein level of some of its binding partners can escape notice in experiments where cells are not confluent.

## Discussion

We have addressed the long-standing confusion regarding the sub-cellular localisation of the HPV16E7 oncoprotein. By employing a quantitative definition of confluence (as defined by number of cells/cm^2^), and using several different cell lines that harbour the viral DNA either as integrated copies in the hosts genome or as extra-chromosomal self-replicating genomes, we have demonstrated that the E7 protein's location within the cells is profoundly influenced by the confluence of the host cell. In sub-confluent cells, E7 is present in both the nucleus and cytoplasm, but in confluent cells the E7 protein becomes predominantly cytoplasmic with increased protein levels. As such, it is possible that much of the conflicting reports on E7's localisation within the cell were caused by analyses that were carried out on cells of different confluence. Cell-based experiments are traditionally carried out at sub-confluence or mid-log phase of growth. However, in the case of HPV16E7, which is normally expressed in keratinocytes of the mucosal epithelium where cells are tightly packed together, assaying cells at confluence is probably a more informative experimental set-up. Consistent with this view, E7 has been found to be predominantly cytoplasmic in some patient biopsies [14], [16].

The switch of E7 localisation was very clear when analysed by immunocytochemistry. However, when sub-cellular fractionation was used together with Western blotting, E7 was found to be predominantly in the cytoplasm in both samples, despite using different protocols for fractionation. This discrepancy of Western blotting detecting cytoplasmic E7 and immunocytochemistry detecting both nuclear and cytoplasmic E7 has also been noted by others. It has been suggested that E7 may leak to the cytoplasmic fraction during cellular fractionation [24], [25]; a suggestion that we find to be very consistent with our comparative analyses. Immunocytochemistry samples on the other hand, were fixed in paraformaldehyde before analysis; preventing any leakage of E7 and permitting the detection of E7 *in situ*. As such we were able to detect E7 both in the nucleus and/or in the cytoplasm, depending on the confluence of the cells. These observations show that the profound influence of analytical procedures on the observations of E7's location is highly significant and must be carefully considered when analysing the E7 protein.

Since a switch in E7 localisation has not been previously described and characterised, we could not benefit from past experience to obtain clues as to the possible molecular causes and mechanisms. Hence we began the characterisation of this switch by considering the known cellular events that are associated with cell confluence. These are cessation of proliferation, arrest within a particular phase of the cell cycle and differentiation; none of which were found to be associated with the cytoplasmic localisation of E7 in confluent cells. Interestingly, Dreier *et al.* [16] reported that they observed the E7 proteins to be present predominantly in large structures encompassing the chromosomes in metaphase and telophase cells. We, on the other hand, did not observe this in cells arrested in mitosis. As can be seen in Figure 7f, nocodazole, which was used to arrest cells in mitosis, did not cause nuclear membrane dissolution or chromosome condensation, which are features of late metaphase and telophase cells. Together these observations suggest that while E7 location within the cell is not altered throughout interphase, it becomes clustered around condensed DNA in the absence of a nuclear membrane. However, as this only occurs when the nuclear membrane is no longer present, it would not be appropriate to conclude that the location of the E7 protein, in respect to the nucleus and cytoplasm, changes as a function of the cell cycle.

Although the general observation of Dreier *et al.* regarding E7 localisation in both the cytoplasm and nuclei of interphase cells is similar to that of ours, the detailed pattern of E7 protein staining between the two studies are somewhat different. Dreier *et al.* observed that cells in interphase had a predominantly diffuse cytoplasmic E7 with a ring structure surrounding the nucleus and faint nuclear E7 microstructures. This difference is most likely brought about by the different antibodies used. While we employed a cocktail of commercially-available E7 antibodies, Dreier *et al.* generated their own rabbit monoclonal antibody against the HPV16E7 protein. With these new reagents, it would be possible in the near future to see the E7 micro-structures in confluent and non-confluent cells expressing E7, which might be more informative. At the moment, we have to conclude that a yet-to-be elucidated mechanism triggered by greater cell-cell contact underlies the shift of E7 from being cytoplasmic and nuclear to almost purely cytoplasmic.

It has been reported that E7 exists as dimers in the nucleus and as spherical oligomers in the cytosol [26]. Hence, it would mean that confluence could favour the formation of E7 oligomers. It would be interesting to study how this is brought about and our observation that confluence can cause E7 to alternate between the two forms could help to delineate the dynamics of the E7 form in the living cell. The fact that E7 exist as dimers in the nucleus may also explain the ease by which it escapes into the cytoplasmic fraction during cellular fractionation. Another interesting observation is that even when E7 is cytoplasmic, it can still be rendered predominantly nuclear by leptomycin B, suggesting that E7 still shuttles between the nucleus and cytoplasm. This is consistent with the ability of E7 to act on its target proteins regardless of its predominant location. On this point, our experiments with SRC-1 also demonstrates that consideration of cellular confluence when experimenting with E7 is pivotal, as failure to do so may allow E7's activity on some cellular proteins to pass un-noticed due to the small magnitude of change observed in sub-confluent cells. A hint of how E7's localisation is connected to its activity can be seen in regards to p130 protein degradation. We observed that the level of this protein rose drastically in confluent NIKS cells, yet in confluent NIKS+HPV16 cells the p130 protein level was very efficiently reduced by E7; which is present almost exclusively in the cytoplasm of these confluent cells. This could indicate that the degradation of p130 is carried out predominantly in the cytoplasm. As it happens, this is indeed the case, as reported Barrow-Laing *et al.* who demonstrated that while p130 can be degraded by HPV16E7 in the nucleus, the bulk activity of p130 degradation by HPV16E7 occurs in the cytoplasm [29]. Interestingly, the low-risk HPV6E7 protein appears to operate differently, whereby it is equally able to degrade p130 in the nucleus as well as in the cytoplasm. When suitable antibodies to HPV6E7 become available, it would be very interesting to analyse the sub-cellular localisation of the HPV6E7 in relation to cell confluence and ascertain if this could be the link to the difference in the way by which it degrades p130. It is equally interesting and important to investigate how other activities of E7 are related to its concentration and location within the cell. Apart from resolving the puzzling question of E7 location in the cell, this work has also raised two interesting questions; the exact pathway that promotes increased levels of E7 and cytoplasmic localisation in confluent cells and the effect of this on other reported E7 activities. The elucidation of these, will undoubtedly teach us more about the activities of this very interesting oncoprotein.

## Materials and Methods

### Cell culture

NIKS provided by Dr Paul Lambert and W12 provided by Dr Margaret Stanley were cultured in F-medium (three parts F-12 Ham:1 part Dulbecco's modified Eagle's medium, 5% fetal calf serum, 24 µg/ml adenine, 8.4 ng/ml cholera toxin, 5 µg/ml insulin, 0.4 µg/ml hydrocortisone and 10 ng/ml epidermal growth factor) with lethally irradiated J2-3T3 [30] feeder cells. J2-3T3 and SiHa were cultured in Dulbecco's modified Eagle's medium supplemented with 10% fetal calf serum. CaSki were cultured in RPMI-1640 medium supplemented with 10% fetal calf serum.

### Recircularisation of HPV16 DNA and NIKS+HPV16

Recircularised HPV16 DNA was generated from the pSPW12 plasmid, provided by Dr Margaret Stanley. Five micrograms of pSPW12 was digested with BamHI to release the full-length HPV16 DNA, followed by a ligation reaction with 2000 U of New England Biolab's T4DNA ligase in a volume of 2 ml at 16°C overnight to recircularise the viral DNA. The recircularised DNA was purified and concentrated using the Qiagen miniprep kit according to the protocol provided. To generate NIKS harbouring episomal HPV16 DNA (NIKS+HPV16) 0.5 million NIKS were transfected with 0.8 µg of recircularised HPV16 DNA and 0.2 µg of pCDNA6A (Invitrogen, Carlsbad, CA) with Effectene transfection reagent (Qiagen). The cells were selected with 8 µg/ml of blasticidin until all untransfected control cells were dead.

### Retrovirus production and NIKS+E7

Retroviral vectors LXSN empty vector control and LXSN16E7, were kindly provided by Dr Denise Galloway. The vectors were transfected into Phoenix A cells (kindly provided by Dr Nolan), and the medium of the cells harvested 48 h later and filtered through a 0.2 µm filter. To generate NIKS expressing E7 alone (NIKS+E7), cells were infected with the retroviruses mixed with polybrene at a concentration of 10 µg/ml and layered onto NIKS. Neomycin was used at 500 µg/ml concentration for selection.

### E7 immunofluorescence microscopy

Coverslips from cell culture were washed in PBS, fixed in 4% paraformaldehyde in PBS for 10 min and washed three times in PBS. The coverslips were then permeabilised for 10 min with 0.1% Triton X-100 (Sigma-Aldrich) in PBS, rinsed once with PBS and blocked in 0.1% BSA (Sigma-Aldrich) in PBST (PBS with 0.1% Tween 20 [Sigma-Aldrich]) for 30 min. The samples were incubated with the E7 primary antibodies (Zymed clone 8C9 and Santa Cruz clone ED17) at 1∶500 in the blocking solution for 4 h, washed 4 times with PBST and incubated for 1.5 h with secondary antibody AlexaFluor488 (A11029, Molecular Probes) at 1∶400 in the blocking solution. The coverslips were washed 4 times with PBST and nuclei were stained with DAPI (Invitrogen) for 20 min. Fixing, permeabilisation, blocking and staining were all at room temperature. Images were captured using a Leica DMI4000 or a Leica SP2 confocal microscope.

### Western blotting

NIKS cells were harvested after the removal of the J2-3T3 feeder layer and lysed with RIPA (150 mM NaCl, 1% TritonX, 0.5% sodium deoxycholate, 0.1% SDS, 50 mM Tris [pH 8.0], 0.005 mM EDTA [pH 8.0]), TEN buffer (50 mM Tris [pH 7.4], 150 Mm NaCl, 1 mM EDTA, 1% NP-40) or for cytoplamic and nuclear fractionation NE-PER nuclear and cytoplasmic extraction reagent kit (ThermoScientific). All buffers were supplemented with a protease inhibitor cocktail (Bio-Rad, Hercules, CA). The protein lysates were quantified by the Bradford method and stored at −70°C. Proteins were separated on 8, 10 or 15% sodium dodecyl sulfate–polyacrylamide gel electrophoresis, transferred onto polyvinylidene difluoride membrane, blocked in 5% milk in PBS–0.5% Tween20 and probed with E7 (Zymed clone 8C9, Santa Cruz clone ED17, 716-325 and NM2), pRb (BD Pharmingen clone G3-245), p130 (Santa Cruz clone C-20) or SRC-1 (Upstate clone 1135) followed by appropriate HRP-linked secondary antibodies.

### Blocking nuclear export with Leptomycin B

Leptomycin B (L2913 Sigma) was added to the culture medium for 3 h at 20 ng/ml. The coverslips were harvested as described and stained for E7. Cells from the same cultures were stained for CyclinB1 (BD Pharmingen clone GNS11) to control for inhibition of nuclear export.

### EdU staining

Cells were treated with EdU (Click-iT EdU kit C10085, Invitrogen) for 25 h to label cells undergoing DNA synthesis. The cells were stained for EdU according to the manufacturer's instructions and the percentage of labelled cells was calculated (>100 cells counted/sample).

### Cell cycle block

Sub-confluent cells were treated with different compounds to arrest the cell cycle at different stages. Mimosine (SigmaAldrich) at 400 uM for 16 h for a G1 block, Thymidine (SigmaAldrich) at 2 mM for 16 h for a S-phase block, Etoposide (SigmaAldrich) at 1 uM for 24 h for a G2 block and Nocodazole (SigmaAldrich) at 40 ng/ml for 24 h for a mitosis block. An untreated sub-confluent and confluent control were also included. Cell cycle blocks were confirmed in parallel by propidium iodide staining analysed by flow cytometry. For propidium iodide staining the cells were trypsinised and fixed in ice-cold 70% ethanol, treated with 0.2 mg/ml of RNAseA (SigmaAldrich) for 15 minutes at 37°C and stained with 40 µg/ml propidium iodide (SigmaAldrich) for 15 minutes.
